# *Blautia luti* reduces neratinib-induced diarrhea in a rat model

**DOI:** 10.1007/s00520-025-09990-w

**Published:** 2025-10-14

**Authors:** Claire P. Vieyra, Micaela J. Quinn, Emma H. Bateman, Hannah R. Wardill, Joanne M. Bowen

**Affiliations:** 1 School of Biomedicine, The University of Adelaide, Adelaide, SA Australia; 2 Supportive Oncology Research Group, Precision Cancer Medicine Theme, South Australian Health and Medical Research Institute, Adelaide, South Australia Australia

**Keywords:** Diarrhea, Neratinib, Microbiome, Probiotics, *Blautia*

## Abstract

**Purpose:**

Neratinib is a pan-human epidermal growth factor receptor (HER) tyrosine kinase inhibitor (TKI) used in the treatment of HER2+ breast cancer. Diarrhea is the most commonly reported toxicity, with the majority experiencing at least some grade of diarrhea. The mechanisms behind neratinib-induced diarrhea are yet to be fully defined, but have been linked to gut microbiome changes, specifically decreased levels of the genera *Blautia*. This study aimed to investigate the efficacy of *Blautia luti (B.luti)* administered as a daily probiotic on neratinib-induced diarrhea, and its effects on the gut microbiome in a well-established rat model.

**Methods:**

Female albino Wistar (AW) rats (*n* = 40) were randomly allocated to groups including; vehicle control (VC), neratinib alone, *B.luti* alone and neratinib + *B.luti* in different schedules (Pre, Pre & Post or Post). Daily oral gavage administration of *B.luti* (10^7^CFU/ml) was given according to corresponding schedules, alongside a 28-day cycle of neratinib (50 mg/kg). Diarrhea was graded daily, and faecal samples collected for gut microbiome analysis at study end. Ileum and colon samples were collected for intestinal analysis. 16S rRNA gene sequencing was performed on faecal samples and H&E performed on intestinal tissue for injury evaluation.

**Results:**

Grade 3 diarrhea was reduced in the Post group when compared to the neratinib alone group (*p* = 0.0122). No significant differences were seen in the tissue injury scores of the ileum or colon. There was no significant change in microbial composition with *B.luti* administration.

**Conclusion:**

This study demonstrated that administration of *B.luti* supplementation, was effective in reducing neratinib-induced diarrhea severity when consumed concurrently. This administration schedule may have a protective role in the intestines through immunomodulation.

## Introduction

Breast cancer has now become the worlds most diagnosed cancer for women [1] with estimates of around 2.3 million new diagnoses annually [2]. Approximately one quarter of these women will be diagnosed with breast cancer that is human epidermal growth factor receptor 2 positive (HER2 +) [3], with overexpression of HER2 associated with tumour cell proliferation, lower treatment efficacy, higher rates of metastases and poorer clinical outcomes [3–5]. The first clinical indication for neratinib, approved in 2017 by the Food and Drug Administration, was in the extended adjuvant setting in HER2 + breast cancer following chemotherapy or trastuzumab-based therapy [6]. Neratinib is a highly potent small molecule tyrosine kinase inhibitor (TKI) that irreversibly binds the intracellular tyrosine kinase domain of HER1, HER2 and HER4 where it inhibits the PI3K and RAS signalling pathways [1, 5, 7, 8] and thus counteracts pro-survival and proliferation signals. In the approval trial for the adjuvant indication, ExteNET (which did not mandate prophylactic anti-diarrheal medication), up to 95% of patients experienced at least some grade of diarrhea during neratinib treatment, with 40% experiencing grades 3–4 diarrhea [9, 10]. Severe diarrhea results in dose reductions, dose interruptions and discontinuation of treatment with unacceptable frequency [11, 12]. Since ExteNET, studies have explored multiple interventions for diarrhea, including loperamide, dose-escalation, Crofelemer, budesonide and Colestipol [13, 14]. Dose escalation has been shown to be effective and is recommended in National Comprehensive Cancer Network guidelines. However, standard treatment options continue to include loperamide prophylaxis which is associated with side effects such as abdominal pain and constipation [15], leaving a gap in care to identify a more tolerable diarrhea intervention.

The mechanisms which drive neratinib-induced diarrhea remain incompletely understood, however, it is believed to be multifactorial in nature [11] involving inflammatory pathways and excess chloride secretion within the intestines [15]. Moreover, the gut microbiome has recently been identified as another potential factor involved in the development of neratinib-induced diarrhea. The gut microbiome plays an important role in the health and wellbeing of an individual, with an influence on immune function, digestion and absorption of nutrients, host metabolism and maintaining intestinal integrity [16–18]. Changes to the gut microbiome have been documented with the use of other TKIs and chemotherapies that cause diarrhea [19], with both clinical and preclinical studies describing gut microbiome changes following neratinib treatment [19–21]. In a study by Secombe et al. (2021), neratinib caused a significant difference in alpha and beta diversity, with the genus *Blautia* being significantly decreased in neratinib-treated rats compared to the control group [20]. A follow-up study, reported that altering the gut microbiome using antibiotics reduced neratinib-induced diarrhea with the genus *Blautia* significantly increased in the group co-treated with antibiotics, indicating a potential research avenue for gut microbiota-targeted therapy [22].

*Blautia* is a member of the *Lachnospiraceae* family which accounts for approximately 50% of the intestinal microbiota [23]. This genus is starting to attract more interest as some species have demonstrated anti-inflammatory effects, potential probiotic properties, antimicrobial effects and the ability to reduce metabolic diseases [23]. *Blautia* is a gram-positive obligate anaerobe commensal bacterium that produces short chain fatty acids (SCFAs), in particular acetate, which act as a fuel source for other bacteria (i.e., cross feeding to promote colonoization resistance), enterocytes and immune cells, making them important for overall gut health and function [23, 24]. Benítez-Páez et al. [25] demonstrated that *Blautia luti (B.luti)* and *B.wexlerae* were negatively correlated with tumour necrosis factor alpha (TNF-α) in obese children compared to control subjects, suggesting depletion in these species may be associated with obesity and insulin resistance [25]. Whilst in the cancer setting, an increased *Blautia* abundance has been shown to reduce morality from graft-vs-host disease and protect against neutropenic fever following bone marrow transplant [26, 27]. This is believed to be due to either direct bacterial interaction or the production of inhibitory factors such as bacteriocins [28]. Although the literature is conflicting regarding *Blautia*, which constitutes 2–8% of the entire gut microbiome in a healthy human gastrointestinal tract [29], it is evident that changes in *Blautia* abundance have a significant impact on host homeostasis. Whether these observed changes in *Blautia* abundance are causal*,* including in neratinib-induced diarrhea, remains unclear. Furthermore, the potential for gut microbiome manipulation to mitigate neratinib-induced diarrhea is unexplored. Considering the evidence for an association between neratinib-induced diarrhea and *Blautia* abundance, it was hypothesized that manipulating the gut microbiome with a *Blautia*-based probiotic would result in decreased diarrhea. As such, this study aimed to determine if the administration of *B.luti* reduces the severity of diarrhea, to evaluate the most effective schedule of *B.luti* administration, to evaluate intestinal protection with *B.luti* administration, and to determine what change occurs in the gut microbiome following *B.luti* administration, using a well-established rat model.

## Methods and materials

### Chemicals and reagents

Neratinib (kindly provided by Puma Biotechnology, USA) was given at 50 mg/kg suspended in 0.5% (w/w) hydroxypropyl methylcellulose (HPMC) (Sigma-Aldrich). *B.luti* (Leibruiz-Intitut DSMZ, Germany) was cultured in yeast extract, casitone, fatty acid and glucose (YCFAG) media (Sigma-Aldrich), under anaerobic conditions (nitrogen (90%), hydrogen (5%) and CO_2_ (5%)), at 37 °C. Purity of culture was confirmed with gram stain. *B.luti* concentration was determined by optical density spectrophotometry (OD_600_) and administered at 1 × 10^7^ CFU/ml in media as per recommendations [30].

### Animals and ethics

This study was approved by The University of Adelaide Animal Ethics Committee (approval number: M-2021–028) and complies with the National Health and Medical Research Council (NHMRC) Australian code for the care and use of animals for scientific purposes 8th edition (updated 2021). Female albino Wistar (AW) rats, sourced from Animal Recourse Centre (ARC) Western Australia, were used for all experiments. Rats were aged between 7 and 8 weeks, with a baseline weight between 170 and 266 g. Rats were housed in pairs on standard bedding with enrichments within individually ventilated cages. Temperature was maintained within 19–23 °C and relative humidity was maintained within the range of 45–65%, with a 12-h light/dark cycle. Rodent chow and water were available ad libitum. If rats were experiencing any treatment related toxicities, they were given soaked rodent chow. Rats were given 7 days to acclimatise to animal house conditions before starting any experiments.

### Experimental design

**Fig. 1:**
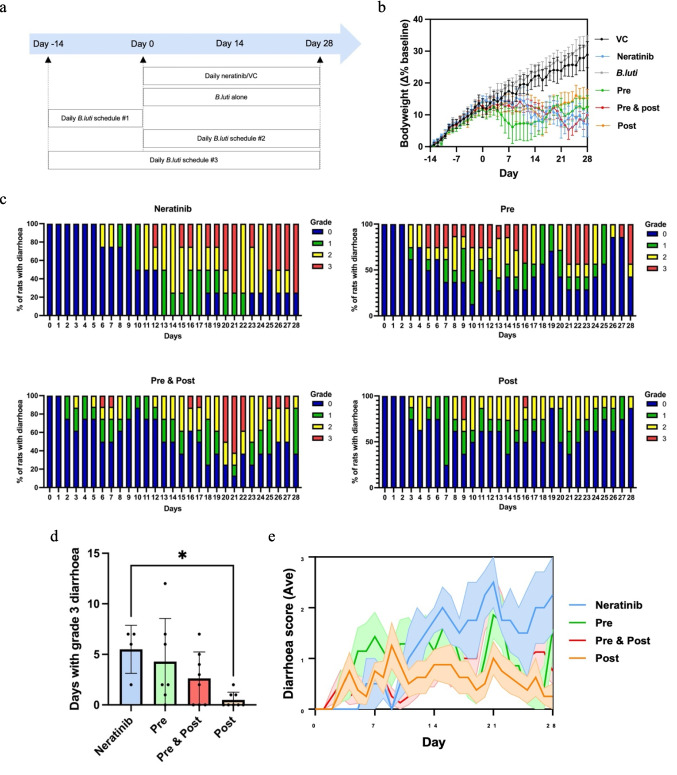
**a** Group allocation and treatment schedules, schedule#1 – Pre, schedule #2 – Post, schedule #3 – Pre & Post. **b** Daily body weight, data shown is mean ± SEM. **c** Daily diarrhea proportions for the neratinib-treated groups. **d** Days with grade 3 diarrhea across the neratinib treated groups. **e** Daily average diarrhea score in neratinib treated groups, data shown is mean ± SEM

Rats (*n* = 40) were randomly allocated to one of six groups (Fig. 1a);

1. Vehicle control (VC); *n* = 4
2. Neratinib alone; *n* = 4
3. *B.luti* alone; *n* = 8
4. Neratinib + *B.luti*; *n* = 8, delivered as 14 days of *B.luti* followed by 28 days of neratinib (**Pre**-schedule)
5. Neratinib + *B.luti; n* = 8, delivered as 28 days of *B.luti* simultaneous with 28 days of neratinib (**Post**-schedule)
6. Neratinib + *B.luti; n* = 8, delivered as 14 days of *B.luti* followed by 28 days of *B.luti* simultaneous with 28 days of neratinib (**Pre & Post**-schedule)

All animals were gavaged every day with either saline/0.5% HPMC or active component, based on their group allocation. Neratinib and VC volumes were adjusted to daily weight changes, and *B.luti* was given at a daily constant 1 ml per animal. All treatments were administered via oral gavage using a soft plastic feeding tube, with the first day being day −14 for *B.luti* and day 0 for neratinib. On day 28, rats were anesthetised with isoflurane, then culled by cardiac blood collection and cervical dislocation.

### Clinical assessment of gut toxicity

Rats were weighed daily and assessed three times daily for diarrhea. Diarrhea was scored on a scale of 0 to 3, according to a well-established system for grading diarrhea in rats [31]. Grade 0, no diarrhea; grade 1, mild diarrhea that is seen as soft stools; grade 2, moderate diarrhea that is seen by way of loose stools along with perineal staining of the fur; grade 3, severe diarrhea that is seen as watery stools along with staining of fur that extends down the hind legs.

### Tissue collection

At day 28, the small intestine and large intestine were removed from rats during necropsy and flushed with sterile chilled phosphate buffered saline (PBS) and weighed. Sections of the ileum and colon were collected and fixed in 10% formalin for embedding in paraffin.

### Histological assessment

Paraffin embedded samples of the distal ileum and proximal colon were cut into 5 μm sections using a rotary microtome before being mounted onto Superfrost glass slides (Menzel-Glasser, Germany). Routine haematoxylin and eosin (H&E) staining was performed, and images taken using a NanoZoomer digital slide scanner (Hamamatsu Photonics). Images were viewed using NanoZoomer Digital Pathology Software (NDP.view 2.8.24). Injury score was assessed using a pre-established system of histopathological criteria [15, 32]. Criteria assessed were villus fusion, villus blunting, epithelial disruption, crypt disruption, immune infiltrate, capillary congestion, oedema and muscularis extern thickening. The colon was assessed using the latter six criteria only. Scoring was performed in a blinded fashion with criteria scored absent = 0, mild = 1 and severe = 2. The injury score is the sum of each criterion, with the distal ileum scored out of a total of 16 and the proximal colon scored out of a total of 12.

### Microbiome analysis

Faecal samples were collected from the anus upon defecation into a sterile tube and stored at − 20 °C before processing. Samples were sent to the Australian Genome Research Facility (AGRF) for DNA extraction and full length 16S rRNA gene sequencing using PacBio long read technology [33].

Target: V1–V9 region.

Forward sequence: 5′GCATC/barcode/AGRGTTYGATYMTGGCTCAG3′

Reverse sequence: 5′GCATC/barcode/RGYTACCTTGTTACGACTT3′

Bacterial diversity profiling was completed on CLC Genomics Workbench 25.0 (QIAGEN). Long read sequences were assembled and trimmed. Trimmed sequences were filtered and sorted by abundance. Using the SILVA reference database (v132, 99% coverage), taxonomy was identified. Data collected; relative abundance at genus level, alpha diversity using the Shannon index, beta diversity which was calculated using permutational multivariate analysis of variance (PERMANOVA) and principal coordinate analysis (PCoA) using Bray–Curtis distances [22].

### Statistical analysis

Data were analysed using GraphPad Prism version 9.2.0. Data are presented as mean ± SEM or mean ± SD, with significance considered *p* $\le$ 0.05. If data passed Anderson–Darling or Shapiro–Wilk test for normality, one-way ANOVA was performed. If normality was not met, non-parametric equivalent tests were performed, namely Kruskal–Wallis.

## Results

### *B.luti* reduced days with neratinib-induced diarrhea

One animal from the Pre group was removed from the study on day 19 due to unexpected weight loss. All other animals maintained body weight throughout the study, although VC and *B.luti* groups had more profound weight gain than groups receiving neratinib, likely associated with diarrhea in the neratinib-treated rats (Fig. 1b). Neratinib was associated with diarrhea of varying grades within a week of starting treatment (Fig. 1c). The administration of *B.luti* led to significantly less days with grade 3 diarrhea in the Post group (0.5 ± 0.3) compared to the neratinib alone group (5.5 ± 1.2) (*p* = 0.0122) (Fig. 1d). Days with grade 1 or 2 diarrhea were not significantly different between groups. Rats in the Pre and the Pre & Post treatment groups experienced no significant reduction to days with diarrhea compared to the neratinib group. No animals from either the VC or the *B.luti* groups experienced any grade of diarrhea (data not shown). The average daily diarrhea grade was not statistically significant between groups (Fig. 1e).

### Diarrhea protection was not associated with histopathological changes

**Fig. 2:**
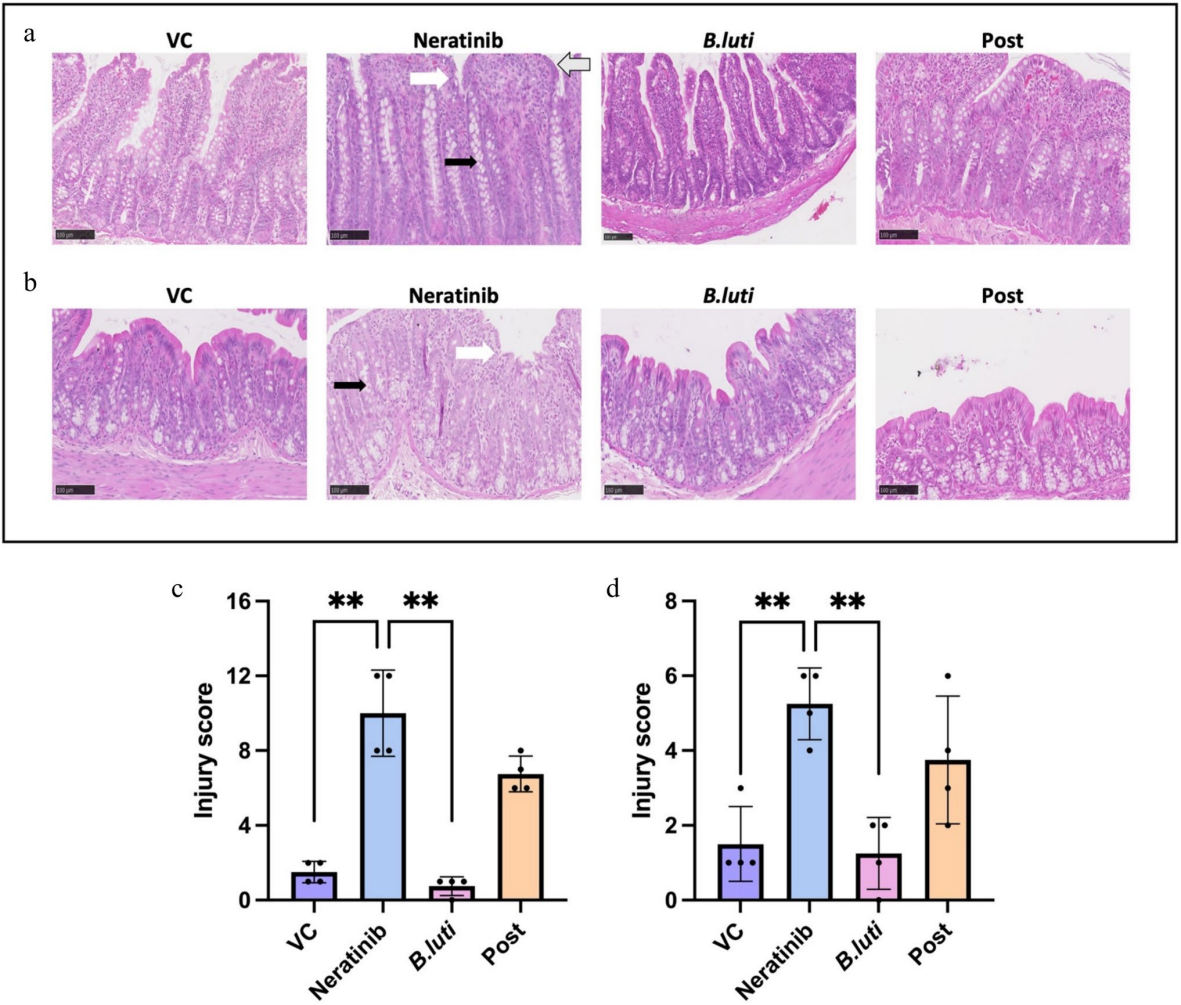
H&E-stained photomicrographs of the **a** distal ileum and **b** proximal colon at × 20 magnification. Black arrow indicates crypt hyperplasia, white arrow indicates enterocyte disruption/flattening, and grey arrow indicates villus blunting and fusion. Injury scores for the **c** distal ileum and **d** proximal colon. Data shown is mean  ± SD, *n* = 4 (group), **p $\le$ 0.01

Given the protection against neratinib-induced diarrhea seen in the Post schedule, this group was focused on for histological changes, with the other two schedules removed from further analysis. Analysis of photomicrographs of the ileum (Fig. 2a) and colon (Fig. 2b) identified typical neratinib related changes, including villous blunting and crypt hyperplasia, as well as surface enterocyte disruption, which was only marginally improved in the Post group. Injury scores for both the distal ileum and proximal colon showed that there were no significant differences in histopathology between the neratinib and Post groups (Fig. 2c and d). However, significant differences in injury scores were noted between VC and neratinib, and neratinib and *B.luti* groups in both the distal ileum (Fig. 2c; *p* $\le$ 0.01), and proximal colon (Fig. 2d; *p* $\le$ 0.01) as expected. As such, *B.luti*-induced diarrhea protection was not associated with significant reduction in histopathology.

### Microbiome analysis

**Fig. 3:**
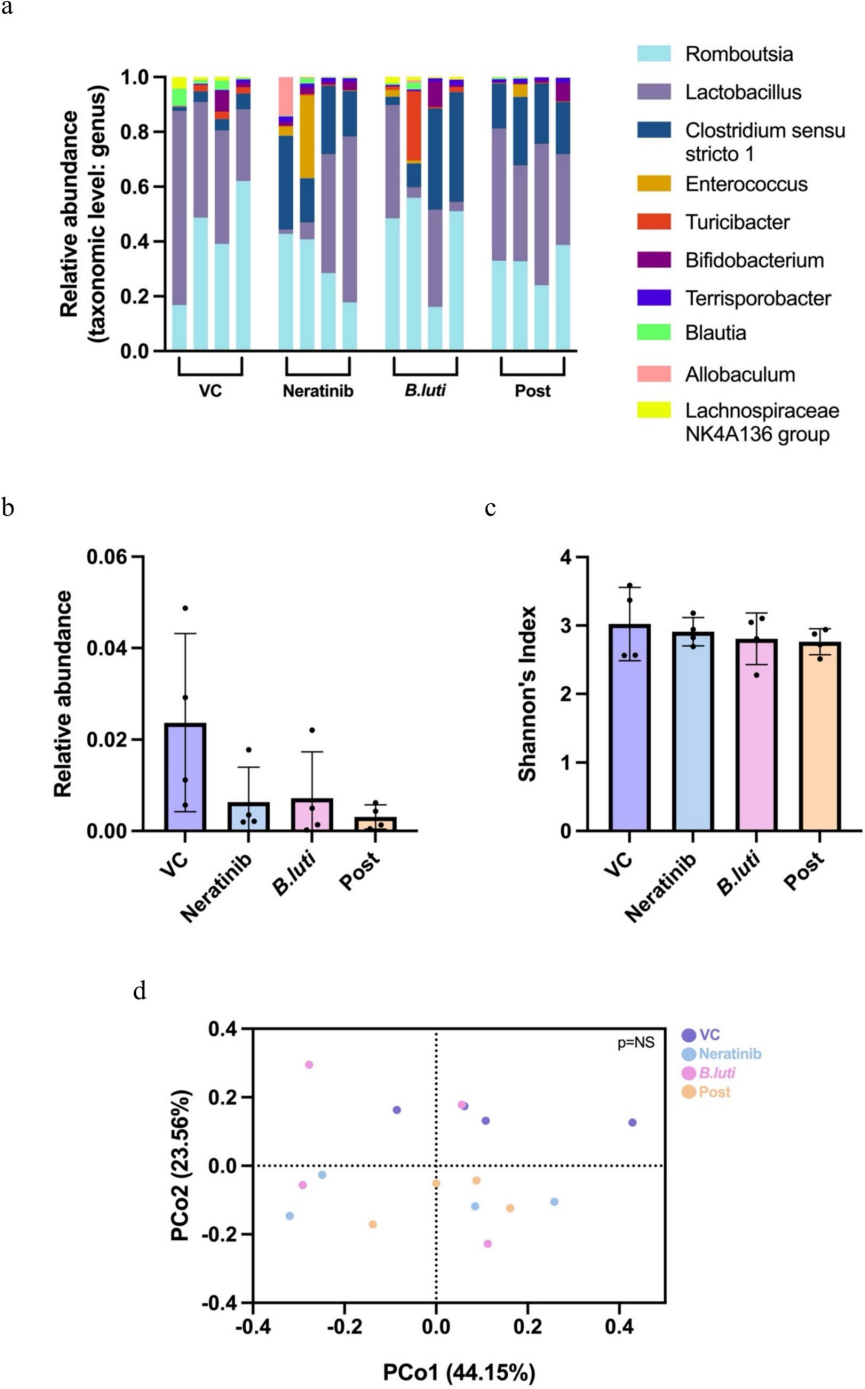
Faecal 16S rRNA gene sequencing; **a** relative abundance of the top 10 most abundant taxa sequenced at the genus level, **b** relative abundance of *Blautia*, data shown is mean  ± SD, **c** alpha diversity using Shannon’s Index, data shown is mean  ± SD, and **d** beta diversity using PERMANOVA and PCoA. *n* = 4 (group)

As the Post schedule was the only *B.luti* group to have a reduced number of days of diarrhea, this group also became the focus of the microbiome analysis, with the other schedules removed from further investigation. Faecal samples collected on day 28 were used for 16S rRNA gene sequencing analysis, and all samples were analysed at the genus level (Fig. 3a–d). If genus level analysis resulted in an uncultured bacterium being identified, it was then analysed at the family level.

The gut microbiome of rats in VC, neratinib, *B.luti* and neratinib + *B.luti* Post groups were found to be similar (Fig. 3a). In terms of the relative abundance of *Blautia*, there was no significant difference observed between the groups (Fig. 3b). Further, at the species level *B.luti* was not identified in any of the samples, noting the limitation of detection using 16S rRNA gene sequencing. Alpha diversity was assessed with Shannon’s diversity index with no significant difference found between the groups (Fig. 3c). Analysis of beta diversity, by PERMANOVA and PCoA, shows no significant shift in overall microbial composition between groups (Fig. 3d).

## Discussion

HER-targeted TKI-induced diarrhea remains a frequent adverse event in breast cancer treatment, and this was the first study to test a targeted probiotic for ability to reduce diarrhea in rats receiving neratinib. It was found that when *B.luti* was given concurrently with neratinib (Post group), the number of days with grade 3 diarrhea was significantly reduced, with no other treatment schedules (i.e., Pre or Pre & Post) producing significant results. There were significant changes in injury scores between neratinib and VC and *B*.*luti *groups in the distal ileum and proximal colon. However, no significant changes in injury scores were observed between neratinib and Post groups in either the distal ileum or proximal colon, indicating that protection from diarrhea was unrelated to observable histopathological changes.

This study extends previous work conducted previously in this model that found neratinib is associated with reduced *Blautia* abundance [20] and elevated *Blautia* levels in rats protected from diarrhea by the gram-negative targeting antibiotic, neomycin [22]. The genus *Blautia* has been associated with probiotic properties, although more research is required at the species level to determine any beneficial effects [23]. Nevertheless, as inflammation represents one of the mechanisms thought to be responsible for neratinib-induced diarrhea, *B. luti* was chosen for its anti-inflammatory potential and SCFA production [23]. The average grade of diarrhea was not significantly reduced by *B. luti*, although it was found that the Post group, where *B. luti* was given concurrently with neratinib, had significantly reduced days with grade 3 diarrhea compared to neratinib alone (4 vs 22 days). Importantly, as none of the *B.luti* supplementation schedules resulted in worse diarrhea than the neratinib alone group, there is no evidence to suggest that *B.luti* is deleterious or increases the risk of injury when consumed prior to treatment initiation. Rather what is clear is that protective effects of probiotic supplementation are confined to the treatment period, suggestive that they are operating in an environment of inflammatory injury to ameliorate further deterioration. This has implications for translation studies that attempt to shift microbiome composition before therapy, with attention perhaps better targeted to on-treatment schedules. Therefore, findings confirm that neratinib-induced diarrhea can be reduced through the introduction of a bacterium as a probiotic and furthers the evidence that gut microbiome influences TKI-associated diarrhea.

Diarrhea is also a major side effect for other cancer therapies, such as chemotherapy, with associated changes to the gut microbiome. One study investigating probiotics in irinotecan-induced diarrhea found that a probiotic cocktail was able to reduce the incidence of diarrhea, an effect that was also timing-dependent of administered probiotic (i.e., before and after chemotherapy) [34]. However, this contradicts the findings of the present study, as Pre or Pre & Post treatments with *B.luti* demonstrated no reduction in days with diarrhea, with a significant improvement only seen in the Post group. Another area of conflict that may have impacted this study is the concentration of bacteria being administered, as there is conflicting information regarding the minimum effective concentration [30]. There is general acceptance that 10^6^ CFU/ml is the minimum effective concentration, however, this number varies greatly across studies with concentrations ranging from 10^6^ to 10^9^ [30, 34, 35]. A study assessing probiotics for the amelioration of chemotherapy-induced intestinal mucositis provides support for the concentration used in the present study (10^7^ CFU) [36]. Despite the dose of *B.luti* in the present being 10^7^ CFU/ml and the significant difference in diarrhea seen in the Post group, higher concentrations may need to be considered for future work to more confidently ascertain the efficacy of *B.luti*.

To better understand *B.luti*’s impact on the gut, sections of the distal ileum and proximal colon were sectioned and stained with H&E. The injury scores for both areas showed no significant difference between neratinib-treated groups. In the distal ileum, there was evidence of villus blunting and fusion across all groups treated with neratinib which were accompanied by changes to the surface epithelium. These findings are validated by other studies where villus fusion and blunting were the main markers of tissue damage in the distal ileum [15, 22, 37]. The proximal colon exhibited no overt evidence of inflammation or oedema, but did show evidence of a loss of surface epithelium and crypt hyperplasia, which is consistent with other studies using neratinib or different TKIs [22, 31, 37], but with varying severity. However, a recent study performed in mice found that neratinib caused severe damage to both the small intestine and colon, with villi integrity lost and severe mucositis in the ileum and jejunum, with moderate colitis in the colon associated with epithelial hyperplasia. It was also reported that there was an increase in proinflammatory cytokines, IL-6 and TNF-α in the ileum, and IL-6 and IL-1β in the colon [38]. Of note, this study used 100 mg/kg of neratinib, considerably higher than the 50 mg/kg that was used in our present study, and a mouse model, which may undergo slightly different degrees of injury in response to the TKI. Although there are conflicting findings of significance in tissue injury, what is consistent is that greater damage to intestinal tissue is located within the ileum, which was also observed in the present study. While variability in injury scores is a limitation of the histological analysis in the present study, there is still a clear lack of differences in tissue histology across the neratinib groups. This indicates that neratinib-induced diarrhea is incompletely related to changes in tissue architecture, with additional factors such as dysregulated electrolyte secretion, mucin alteration and altered water balance contributing to symptoms. In an ex vivo study, Lysyy et al. (2019) demonstrated that luminal exposure to neratinib produced a marked increase in fluid secretion along the length of the ileum and colon which could explain aspects of the diarrhea peominant-phenotype [39].

Unexpectedly, analysis of the gut microbiome found low levels of *Blautia* in faecal samples, and no significant difference in alpha or beta diversity between groups. The overall abundance of *Blautia* was lower than expected, which is unusual for a genus that normally occupies 2–8% of the human gut microbiome [29]. There were no significant changes in the relative abundance of *Blautia* observed between VC and neratinib groups, which is in stark contrast to previous studies in this model. Previous studies reported much higher relative abundance of *Blautia* along with highly significant differences in the relative abundance of *Blautia* between control and neratinib treated rats in caecal samples and antibiotic treated rats in faecal samples [20, 22]. Faecal microbiome analysis also revealed no significant changes to *Blautia* abundance with the *B.luti* intervention. However, analysis of faecal contents effectively samples only the luminal microbiome, and there has been some evidence to suggest *Blautia* preferentially colonises the mucosal layers of the gut, with higher levels found in mucosal biopsies compared to faecal samples [40]. It has also been demonstrated that bacterial colonisation differs with location in the intestinal tract due to pH and oxygen levels [41]. Hence, analysis of caecal samples or indeed small intestinal lavage may yield different results. In addition, most studies suggest that TKI-induced diarrhea is a small intestinal phenomenon, and thus caecal contents may be more representative of the small intestinal microbiome and any protection provided by the *B.luti* intervention. Additionally, 16S sequencing is also very limited at the species level, making it difficult to determine anything past the genus level. Therefore, shotgun metagenomics should also be considered in future explorations of *B.luti* as it will provide a more focused and detailed analysis of the faecal microbiome and potential alternations in function. *B.luti* administration may not have resulted in reducing diarrhea by dominant colonisation of the gut but may have acted in other ways to support other beneficial bacteria. *Blautia* is an acetogen [23, 42] which not only can be used by other bacteria for nutrients to potentially increase their abundance by way of cross feeding, but also as an acetogen they can change the acidity of the surrounding environment and discourage the growth of pathogens [23]. Based on a study showing that *B.producta* was able to inhibit the growth of VRE, it is believed that *Blautia* may have the ability to produce bacteriocins that give them antibiotic properties [23, 28]. With a low number of studies having researched *B.luti*, it is difficult to say how it impacts the gut specifically and future work should focus on measuring metabolites in addition to sequencing data.

In conclusion, this study was the first investigation of *B.luti* as a possible probiotic to reduce the number of days with neratinib-induced diarrhea. The significant reduction in days with grade 3 diarrhea seen with the schedule of *B.luti* concurrent with neratinib treatment, suggests that the gut microbiome is a potential target for new anti-diarrheal treatments. Further investigation into the mechanisms of neratinib-induced diarrhea is required because although it has been shown in some studies that neratinib is associated with a changed gut microbiome, the causality of this relationship remains unclear.

## Data Availability

Not available.
